# Effects of Warfarin on the Risks of Mortality, Acute Heart Failure, and Infection Resolution in Patients With Infective Endocarditis: A Target Trial Emulation

**DOI:** 10.1161/JAHA.125.041965

**Published:** 2025-07-17

**Authors:** Teddy Tai Loy Lee, Chengsheng Ju, Sunny Ching Long Chan, Oscar Hou In Chou, Jeffrey Shi Kai Chan, Sharen Lee, Tong Liu, Shuk Han Cheng, Yuhui Zhang, Bernard Man Yung Cheung, Abraham Ka‐Chung Wai, Li Wei, Gary Tse

**Affiliations:** ^1^ Department of Emergency Medicine, School of Clinical Medicine The University of Hong Kong Hong Kong China; ^2^ Cardiovascular Pharmacology Unit, Cardiovascular Analytics Group PowerHealth Research Institute Hong Kong China; ^3^ Research Department of Practice and Policy, School of Pharmacy University College London London United Kingdom; ^4^ Institute of Cardiovascular Science, University College London London United Kingdom; ^5^ Division of Clinical Pharmacology, School of Clinical Medicine The University of Hong Kong Hong Kong China; ^6^ Tianjin Institute of Cardiology, The Second Hospital of Tianjin Medical University Tianjin China; ^7^ Department of Biomedical Sciences City University of Hong Kong Hong Kong China; ^8^ Heart Failure Center, State Key Laboratory of Cardiovascular Disease Fuwai Hospital, National Center for Cardiovascular Diseases, Chinese Academy of Medical Sciences and Peking Union Medical College Beijing China; ^9^ School of Nursing and Health Sciences Hong Kong Metropolitan University Hong Kong China

**Keywords:** anticoagulation, causal inference, infective endocarditis, trial emulation, Valvular Heart Disease, Infectious Endocarditis, Mortality/Survival

## Abstract

**Background:**

Infective endocarditis (IE) can be complicated by acute heart failure and bacteremia, which can account for increased mortality. The role of anticoagulation with warfarin in IE is controversial. This study aimed to study the effects of anticoagulation with warfarin on survival in patients with IE, through reducing the risks of thromboembolism and possibly shortening infection time.

**Methods:**

This was a retrospective population‐based cohort study using the Clinical Data Analysis and Reporting System from Hong Kong. Patients diagnosed with IE between January 1, 1997 and August 31, 2020 were identified using *International Classification of Diseases, Ninth Revision* (*ICD‐9*) codes. A target pragmatic trial was emulated using the observational data with cloning‐censoring‐weighting approach, comparing the treatment effect of initiation warfarin within 14 days versus no warfarin on the risk of all‐cause mortality, acute heart failure, and achieving negative blood culture in patients with IE. Pooled logistic regression was applied to estimate 12‐week survival or cumulative incidence differences and risk ratios (RRs).

**Results:**

A total of 5121 patients with IE with an average age of 55.7 years (SD:18.9) were included. Warfarin use was associated with lower risks of all‐cause mortality with 12‐week survival difference of 6.5% (95% CI, 2.6%–9.9%) and RR of 0.72 (95% CI, 0.57–0.88) and a greater benefit of achieving negative blood cultures with 12‐week cumulative incidence difference of 11.4% (95% CI, 5.4%–16.5%) and RR of 1.13 (95% CI, 1.06–1.20) but similar risks of acute heart failure (RR, 1.07 [95% CI, 0.87–1.30]).

**Conclusions:**

Patients with IE initiating warfarin had significantly lower risk of mortality with potential benefits on achieving negative blood cultures, suggesting benefit in infection resolution but a similar risk of acute heart failure.

**Nonstandard Abbreviations and Acronyms**
AHF: acute heart failure
IE: infective endocarditis

**Clinical Perspective**

**What Is New?**

- Initiating warfarin within 14 days of diagnosis of infective endocarditis may lead to a significant reduction in mortality risk.
- Warfarin use may also aid infection resolution as evidenced by achievement of negative blood cultures.

**What Are the Clinical Implications?**

- This study supports the potential use of anticoagulation therapy in the treatment of infective endocarditis.

Acute heart failure (AHF) is a common complication of infective endocarditis (IE).^1^ In IE, AHF is caused by valvular regurgitation due to valvular damage by the developing vegetation, and commonly warrants cardiac surgery as treatment. In severe cases, AHF is presented as cardiogenic shock, which presents as tissue hypoperfusion and hemodynamic instability. AHF contributes to poor prognosis in patients with IE and is a cause of the high mortality rate in patients diagnosed with IE.^2^ Currently, international guidelines recommend cardiac surgery as treatment for patients with IE presenting with severe AHF.^3^, ^4^ However, not all patients are candidates for cardiac surgery as it is complicated by several factors, the most important of which are neurological complications. Guidelines recommend that cardiac surgery is delayed in patients suffering from intracranial hemorrhage or stroke. Cardiac surgery also poses risks of arrhythmia and formation of blood clots or introduction of new infection. As such, there are needs for treatment that potentially reduce the risks of developing AHF.

Alternatively, IE may support the persistence of bacteremia, where the vegetation acts as a stable reservoir for continuous release of bacteria to the bloodstream. This may be associated with increased mortality. Treatment of infection is thus limited to lengthy antibiotic regimens, ranging from 4 to 6 weeks of treatment, until which infection resolution is expected in patients with IE.^5^ However, the clinical utility of extended use of antibiotics is limited by the acuity of aggressive IE.

Recent studies have suggested warfarin may have a beneficial role in the management of IE.^6^, ^7^ As AHF is induced by valvular damage originating from vegetation growth, the use of warfarin may exhibit protective effects in reducing vegetation size by preventing the recruitment of clotting factors to heart, thus slowing vegetation growth and minimizing subsequent valvular damage. Patients prescribed with warfarin have better prognosis such as decreased mortality.^6^ However, the cause of decreased mortality associated with warfarin use remains unknown. It is hypothesized that warfarin can decrease IE mortality through a reduction in AHF events. Another mechanism would be hastening dissolution of the vegetation bacterial reservoir, leading to prompt resolution of infection. Although anticoagulation is commonly applied in the management of thromboembolism, its application remains controversial among patients with IE because of the lack of randomized controlled trials and its potential side effects.^8^ As such, current guidelines have provided few recommendations on the use of anticoagulants among patients with IE, such that there is a need for further evidence regarding the benefits of warfarin on IE.^9^, ^10^ To the best of our knowledge, this is the first target trial emulation study evaluating the effects of warfarin initiation in a population‐based cohort of adult patients with IE.

## METHODS

### Data Source

The data that support the findings of this study are available from the corresponding author upon reasonable request. Pseudonymized data were retrieved from the Clinical Data Analysis and Reporting System, a territory‐wide electronic health care records system managed by the Hong Kong Hospital Authority, which serves an estimated 90% of the population in Hong Kong. The Clinical Data Analysis and Reporting System has previously been used extensively to conduct large population‐based studies,^11^, ^12^ including those on anticoagulation use.^13^ Informed consent was not required due to anonymity of study data.

### Ethics Approval and Consent to Participate

This study was approved by The University of Hong Kong/Hospital Authority Hong Kong West Cluster Institutional Review Board (UW‐20‐250) and The Joint Chinese University of Hong Kong Hospital Authority New Territories East Cluster Clinical Research Ethics Committee (2019.338).

### Study Design

This is a population‐based cohort study applying the target trial emulation framework (ie, an observational study emulating a pragmatic clinical trial) on patients with IE using the Clinical Data Analysis and Reporting System database. The cloning‐censoring‐weighting approach was used to emulate a target pragmatic trial comparing the treatment effect of warfarin on all‐cause mortality, AHF, and achieving negative blood cultures indicating infection resolution in patients with IE. The specification and emulation of the target trial are presented in Table S1.

### Eligibility Criteria

Patients aged 18 years and over with an IE diagnosis (*International Classification of Diseases, Ninth Revision* (*ICD‐9*) diagnosis codes of 424.9, 421, 93.2, 36.42) between January 1, 1997 and August 31, 2020 were eligible for this study. The baseline time (*T*_0_) was defined as the date of the IE diagnosis during the study period. We excluded patients with previous heart failure or valvular replacement or with the use of warfarin or any other anticoagulants (rivaroxaban, dabigatran, apixaban, edoxaban, enoxaparin, fondaparinux, heparin) within 30 days before the IE diagnosis.

### Treatment Strategies

We compared the treatment strategies of receiving warfarin treatment 14 days within the IE diagnosis to not starting warfarin treatment after the IE diagnosis. The 14‐day (2‐week) period after the IE diagnosis was used as a grace period to screen for the initiation of warfarin after IE. Indications of warfarin initiation may include prosthetic valve use, venous thromboembolism, or prevention of embolic stroke or renal infarction secondary to IE.

### Follow‐Up Period and Outcomes

The follow‐up commenced from *T*_0_ until an occurrence of outcome (AHF or negative blood culture), death, or 12 weeks after *T*_0_. The study outcomes were all‐cause mortality, AHF, and negative blood culture. AHF was defined as (1) use of inotropes and vasopressors (noradrenaline, dopamine, adrenaline, phenylephrine, vasopressin, or dobutamine); (2) use of intra‐aortic balloon pump (*ICD‐9* procedure code 37.61); (3) use of intravenous furosemide; and (4) use of extracorporeal membrane oxygenation (*ICD‐9* procedure code 39.65). The analysis on achieving negative blood culture was nested in patients who had at least 1 blood culture test after the IE diagnosis.

### Covariates

Baseline covariates included age, sex, calendar year of the IE diagnosis (1997–1999, 2000–2002, 2003–2005, 2006–2008, 2009–2011, 2012–2014, 2015–2017, 2018–2020); Charlson Comorbidity Index; individual comorbidities within past 2 years before *T*_0_ including hypertension, atrial fibrillation, diabetes, intracranial hemorrhage, chronic kidney disease, vascular disease; medications within past 30 days including angiotensin‐converting enzyme inhibitors, angiotensin II receptor blockers, antiplatelets, beta blockers, nonsteroidal anti‐inflammatory drugs; histamine‐2 receptor antagonists, proton pump inhibitors, and selective serotonin reuptake inhibitors; pathogen identified from blood culture including Staphylococci, Streptococci, Enterococci, and the *Haemophilus* species, *Aggregatibacter* species, *Cardiobacterium hominis*, *Eikenella corrodens*, and *Kingella kingae* group.

We considered the role of time‐varying covariates that may affect management of IE and coagulation risk during admission, which included cardiovascular surgery, heparin treatment, direct oral anticoagulant treatment, as well as all other medications that were measured at the baseline, which were updated at weekly intervals since the IE diagnosis.

All the aforementioned covariates were included in the weighting models, and the same set of covariates was applied across all 3 outcomes. A directed acyclic graph describes the causal structure between study exposure, outcomes, and confounders in Data S1.

### Statistical Analysis and Emulation of the Target Trial

To emulate a target trial, we applied the cloning‐censoring‐weighting approach with a grace period of 14 days (2 weeks) to screen for the receipt of warfarin treatment.^14^, ^15^ We created a data set with 2 copies of each eligible individual (ie, cloning) and assigned each of the replicates to 1 of the treatment strategies at the start of follow‐up (*T*_0_—the IE diagnosis). Thereafter, at weekly intervals, we assessed whether replicates adhered to their assigned treatment strategy; replicates were censored if and when their actual treatment deviated from their assigned treatment strategy, thereby ensuring that replicates followed their assigned strategy. That is, if a replicate was assigned to receiving warfarin treatment, but did not receive warfarin by the end of the 14‐day period, they would be censored at that point. Conversely, if a replicate is assigned to no warfarin treatment, but received warfarin at any time during the follow‐up, they would be censored at that point. Any outcomes that occurred during the grace period before artificial censoring were included in both treatment arms to avoid immortal time bias. To adjust for the potential selection bias induced by this censoring, each individual received a time‐varying inverse probability weight. The denominator of the weight was the probability that a replicate remained on the assigned treatment strategy conditional on baseline and time‐varying characteristics. The weights created 2 pseudo‐populations in which treatment was independent of measured confounding or prognostic factors. We estimated the time‐varying weights by fitting logistic models for the weekly probability of remaining uncensored, including variables for time in week (in its linear and quadratic terms), and the baseline and time‐varying covariates. To avoid undue influence of outliers, weights were truncated at the 99th percentile. Further details of the cloning‐censoring‐weighting approach are available in Data S2.

In the analysis of the AHF outcome, we considered death as the competing risk event by censoring death in a cause‐specific model. Because censoring due to death may introduce selection bias, we additionally calculated an inverse probability of death weight, based on the probability of a patient being alive during each time interval, using a similar method as for the inverse probability of censoring weight. Thereby, to estimate the cause‐specific risk of AHF we created a pseudo‐population in which it was as if any death has not occurred.^16^ The final weight is the product between the inverse probability of censoring weight and death weight.

In the analysis of the negative blood culture outcome, we selected patients who had at least 1 blood culture test after the IE diagnosis. To account for the selection bias, we calculated the inverse probability weight based on the probability of having a blood culture test conditional on the baseline covariates.^17^ Similar to the analysis on the AHF outcome, we address the competing risk from death using an inverse probability of death weight. The final weight for the blood culture outcome was the product of inverse probability weights of blood culture test, death, and censoring due to treatment deviation.

We estimated the effect of warfarin on study outcomes using a weighted pooled logistic regression, including an indicator for treatment strategy, time in week (in its linear and quadratic terms), and their interactions to allow for nonproportional hazards. Pooled logistic regression is a discrete‐time hazard model that is commonly used in the causal survival analysis.^18^, ^19^ We used the pooled logistic regression model to estimate the probability of outcome occurrence during each follow‐up time interval (weekly) under each treatment arm, and the overall cumulative survival probability from the outcome over the entire follow‐up time,^20^ which was used to plot the survival or cumulative incidence curves. To differentiate the negative end points (mortality and AHF) and positive end point (achieving negative blood culture test), we presented survival curves for the negative end points and cumulative incidence curve for the positive end point. Then, 12‐week survival probability or cumulative incidence, survival or cumulative incidence differences, and risk ratios were presented accordingly with pointwise 95% CIs using a nonparametric bootstrap of 500 samples. We also approximated hazard ratios (HRs) from a Cox regression using odds ratios from the pooled logistic regression and 95% CIs with the robust variance estimator, given that the outcome is rare during each follow‐up interval.^21^

Baseline characteristics were presented as median (interquartile range) for continuous variables and as numbers (%) for categorical variables. Standardized mean difference was used to evaluate the differences in baseline variables between groups. A standardized mean difference <0.2 was considered as good balance between groups. Findings were considered to be statistically significant when the 95% CIs for risk on a relative scale did not cross 1 or when the 95% CI for risk difference on an absolute scale did not cross zero.

All analyses were performed with either R version 4.3.1 (The R Foundation) or SAS version 9.4 (SAS Institute Inc).

### Sensitivity Analysis

First, we truncated the weight at 99.5th percentile rather than 99th, to evaluate the impact of extreme weights and weight truncation. Second, we changed the grace period from 2 weeks to 1 week and 3 weeks, to evaluate the impact of delayed treatment initiation. Third, we used doubly robust estimation models as the outcome models, in which the baseline covariates were adjusted in the model on top of the weights. Fourth, we used an alternative definition for AHF by excluding postoperative use of inotropes and vasopressors or any use of inotropes and vasopressors. Fifth, we stratified all analyses by calendar period of 1997 to 2008 and 2009 to 2020.

## RESULTS

### Patient Characteristics

**Figure 1:**
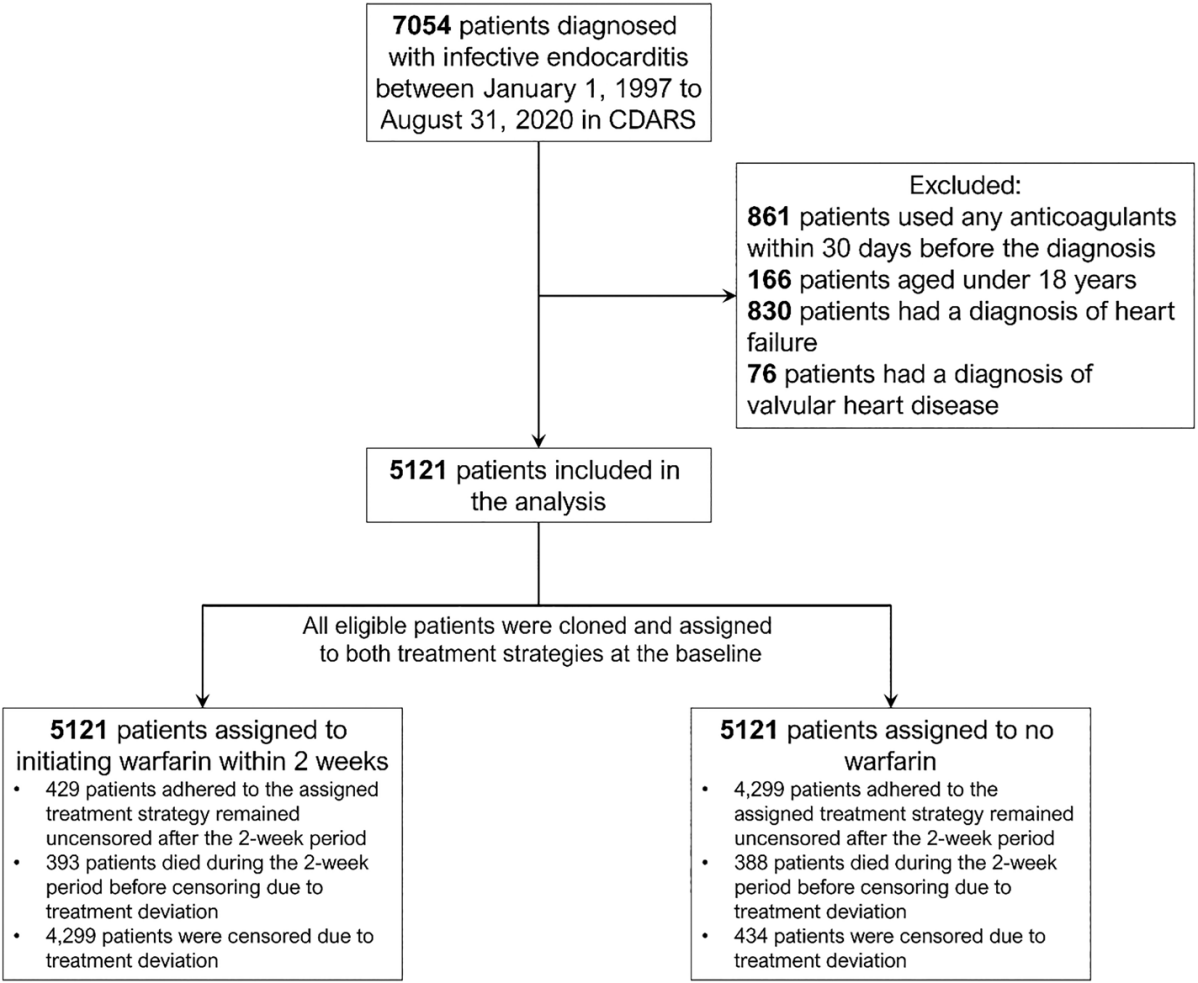
Selection of patients with infective endocarditis from CDARS for the trial emulation. CDARS indicates Clinical Data Analysis and Reporting System.

A total of 5121 patients who met the eligibility criteria were included in the analysis (Figure 1). The mean age (±SD) of this study cohort was 55.7 years (±18.9). All patients were assigned to both initiation warfarin within 14 days and no warfarin treatment strategies. Of these, 486 (9.5%) patients initiated warfarin within the 14‐day grace period. After the grace period, 429 (9.1%) patients who had initiated warfarin and 4299 (90.9%) patients who had not initiated warfarin remained in the cohort (Table 1). The inverse probability weights were calculated separately for warfarin and no warfarin arms and for each study outcome, the model coefficients for the inverse probability weight for receiving warfarin treatment for each study outcome are presented in Table S2, and the distributions of the weights for each study outcome were presented in Table S3. The baseline characteristics before and after applying the inverse probability of censoring weights method after censoring over the grace period were presented in Table S4, all variables were balanced after weighting. The frequencies of time‐varying covariates by treatment groups at all time points were also presented, which can be found in Table S5.

**Table 1:** Baseline Characteristics of the Study Cohorts, Before and After the Grace Period

	Baseline	After the grace period	After the grace period
	All patients (n=5121)	Warfarin (n=429)*	No warfarin (n=4299)*
Age, y (SD)	55.7 (18.9)	56.7 (16.5)	55.5 (19.2)
Male sex (%)	3420 (66.8)	252 (58.7)	2881 (67.0)
Charlson Comorbidity Index (SD)	1.6 (2.1)	1.7 (1.9)	1.6 (2.1)
Calendar y (%)			
1997–1999	827 (16.2)	2 (0.3)	751 (17.5)
2000–2002	664 (13.0)	47 (7.6)	571 (13.3)
2003–2005	596 (11.6)	54 (9.7)	505 (11.8)
2006–2008	601 (11.7)	55 (9.8)	505 (11.8)
2009–2011	588 (11.5)	64 (12.0)	470 (10.9)
2012–2014	574 (11.2)	50 (9.6)	471 (11.0)
2015–2017	657 (12.8)	79 (12.9)	532 (12.4)
2018–2020	614 (12.0)	78 (13.6)	494 (11.5)
Comorbidities (%)			
Hypertension	698 (13.6)	61 (14.2)	591 (13.8)
Atrial fibrillation	284 (5.6)	90 (20.1)	169 (3.9)
Diabetes	455 (8.9)	33 (7.7)	395 (9.2)
Intracranial hemorrhage	110 (2.2)	9 (2.1)	89 (2.1)
Chronic kidney disease	217 (4.2)	11 (2.6)	189 (4.4)
Vascular disease	31 (0.6)	2 (0.5)	25 (0.6)
Recent medications (%)			
Angiotensin‐converting enzyme inhibitors	385 (7.5)	55 (12.8)	310 (7.2)
Angiotensin‐II receptor blocker	110 (2.2)	17 (4.0)	89 (2.1)
Beta blockers	483 (9.4)	73 (17.0)	377 (8.8)
Antiplatelets	540 (10.5)	73 (17.0)	422 (9.8)
Nonsteroidal anti‐inflammatory drugs	803 (15.7)	94 (21.9)	642 (14.9)
Histamine‐2 receptor antagonists	668 (13.0)	83 (13.6)	527 (12.3)
Proton pump inhibitors	557 (10.9)	55 (12.8)	448 (10.4)
Selective serotonin receptor inhibitors	43 (0.8)	4 (0.9)	38 (0.9)
Pathogen from blood culture (%)			
Staphylococci	1025 (20.0)	42 (9.8)	824 (19.2)
Streptococci	701 (13.7)	30 (7.0)	628 (14.6)
Enterococci	101 (2.0)	7 (1.6)	87 (2.0)
*Haemophilus* species, *Aggregatibacter* species, *Cardiobacterium hominis*, *Eikenella corrodens*, and *Kingella kingae*	35 (0.7)	0 (0)	35 (0.8)

* These numbers do not add up to the total number of patients as some patients who experienced study outcomes or administrative censoring over the grace period did not remain in the cohort after 2 weeks.

### Warfarin Treatment and All‐Cause Mortality

**Figure 2:**
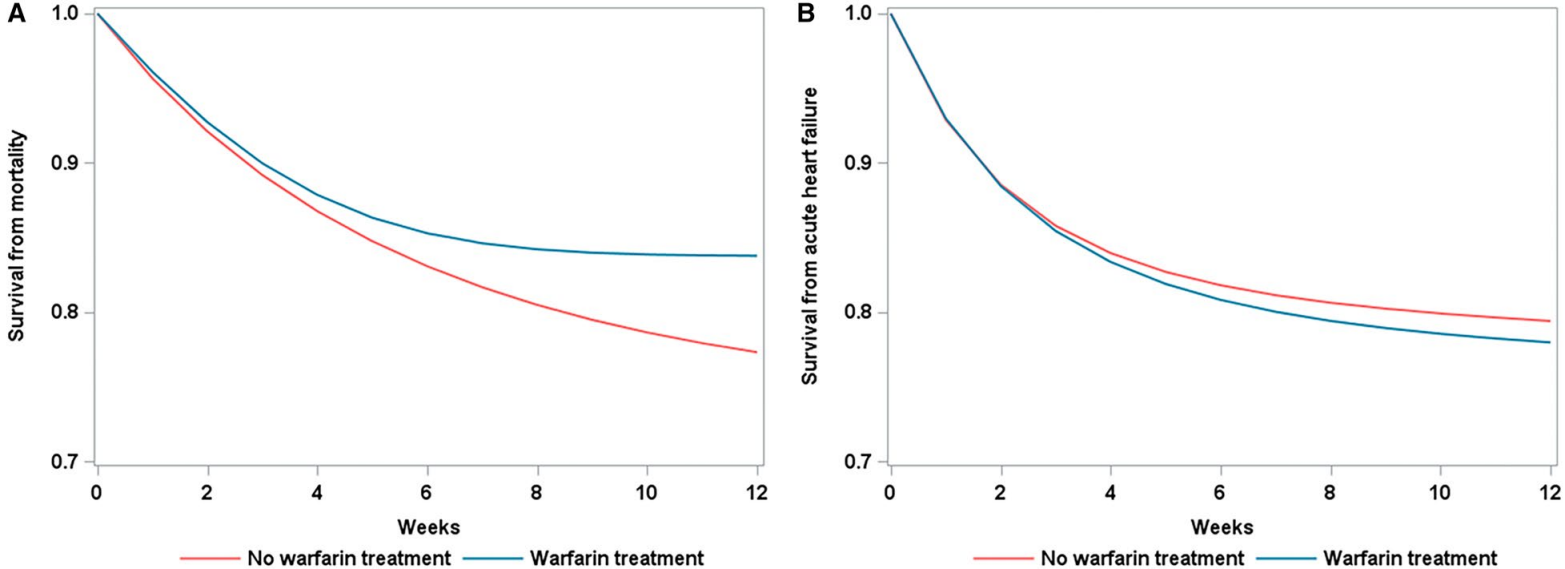
Weighted survival curves under warfarin versus no warfarin treatment. **A**, from all‐cause mortality; **B**, from acute heart failure.

There were 424 and 1005 deaths amongst the initiating warfarin and no warfarin treatment strategy arms, respectively. The 12‐week survival probability from all‐cause mortality was 83.8% (95% CI, 80.7%–86.9%) for warfarin arm, and 77.3% (95% CI, 75.6%–79.0%) for no warfarin arm. The 12‐week survival difference was 6.5% (95% CI, 9.9%–2.6%) and 12‐week risk ratio (calculated as the ratio of 1‐survival probability) was 0.72 (95% CI, 0.57–0.88) for warfarin versus no warfarin (Figure 2A). The corresponding HR, as the average risk over the follow‐up period, was 0.72 (95% CI, 0.56–0.89) (Table 2).

**Table 2:** Twelve‐Week Absolute Risks, Risk Differences, Risk Ratios, and Hazard Ratios for All‐Cause Mortality, AHF, and Negative Blood Culture Under Initiating Warfarin and Versus No Warfarin

Treatment	No. of patients	No. of patient‐weeks	No. of outcomes	12‐week absolute risk (%) (95% CI)	12‐week risk difference (%) (95% CI)	12‐week risk ratio (95% CI)	Hazard ratio (95% CI)
All‐cause mortality							
Warfarin	5121	14 140	424	16.2 (13.1 to 19.3)	−6.5 (−9.9 to −2.6)	0.72 (0.57 to 0.88)	0.72 (0.56 to 0.89)
No warfarin	5121	46 105	1005	22.7 (21.0 to 24.4)	Reference	Reference	Reference
AHF							
Warfarin	5121	12 837	592	22.0 (17.8 to 26.5)	1.4 (−2.6 to 5.9)	1.07 (0.87 to 1.30)	1.07 (0.88 to 1.31)
No warfarin	5121	42 661	889	20.6 (19.0 to 22.2)	Reference	Reference	Reference
Negative blood culture							
Warfarin	3662	5155	2829	96.4 (90.9 to 99.9)	11.4 (5.4 to 16.5)	1.13 (1.06 to 1.20)	1.16 (1.06 to 1.27)
No warfarin	3662	8275	3201	85.0 (81.9 to 88.4)	Reference	Reference	Reference

AHF indicates acute heart failure.

### Warfarin Treatment and AHF

There were 592 and 889 cases of AHF under the initiating warfarin and no warfarin treatment strategies, respectively. The patients with AHF were identified based on the presence of any of the 4 components: extracorporeal membrane oxygenation (n=19), intra‐aortic balloon pump (n=50), use of inotropes or vasopressors (n=612), or intravenous furosemide (n=537). The 12‐week survival probability from AHF was 78.0% (95% CI, 73.5%–82.2%) for the warfarin and 79.4% (95% CI, 77.8%–81.0%) for no warfarin. The 12‐week survival difference was −1.4% (95% CI, −5.9% to 2.6%) and 12‐week risk ratio was 1.07 (95% CI, 0.87–1.30) for warfarin versus no warfarin (Figure 2B). The corresponding HR was 1.07 (95% CI, 0.88–1.31) (Table 2).

### Warfarin Treatment and Achieving Negative Blood Culture

**Figure 3:**
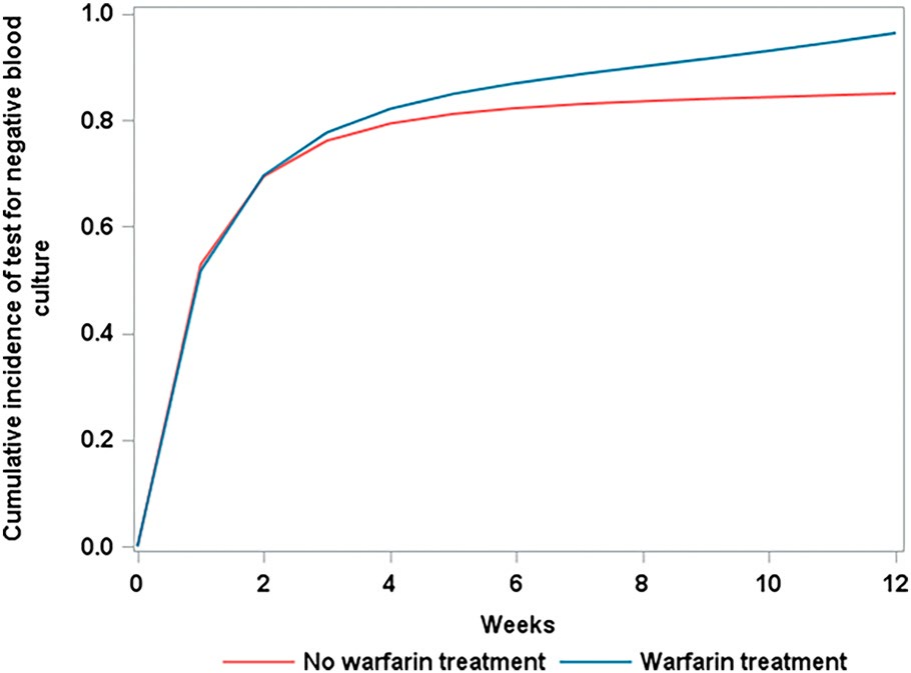
Weighted cumulative incidence curves under warfarin versus no warfarin treatment for test for negative blood culture.

In the analysis on negative blood culture, the 12‐week absolute risk of negative blood culture was 96.4% (95% CI, 90.9%–99.9%) for the warfarin and 85.0% (95% CI, 81.9%–88.4%) for no warfarin. The 12‐week difference in cumulative incidence was 11.4% (95% CI, 5.4%–16.5%) and 12‐week risk ratio was 1.13 (95% CI, 1.06–1.20) for warfarin versus no warfarin (Figure 3). The corresponding HR was 1.16 (95% CI, 1.06–1.27) (Table 2). Therefore, warfarin use is associated with higher rates of recovery from bacteremia.

### Sensitivity Analysis

In sensitivity analysis with varied weight truncation, grace period, doubly robust estimation, or alternative definition of AHF, the estimates were consistent with the main analysis. The 12‐week risk ratio ranged from 0.71 to 0.76 for all‐cause mortality, 0.87 to 1.13 for AHF, and 1.05 to 1.14 for negative blood culture (Tables S6–S10). In the stratified analysis by calendar time, warfarin was associated with a lower risk of mortality between 2009 to 2020 (HR, 0.55 [95% CI, 0.40–0.74]) but not between 1997 and 2008 (HR, 0.90 [95% CI, 0.65–1.24]), although the 95% CIs overlapped; warfarin was associated with a higher rate of negative blood culture test between 1997 and 2008 (HR, 1.25 [95% CI, 1.06–1.47]) but not between 2009–2020 (HR, 1.05 [95% CI, 0.97–1.14]) because the 12‐week rate of achieving negative blood culture was high in both groups (warfarin, 99.4%; no warfarin, 96.5%). No association with AHF was observed in both strata (Table S11).

## DISCUSSION

This is the first target trial emulation evaluating the risk and benefits of warfarin initiation in patients with IE. Although we found that patients with IE taking warfarin had a significantly lower risk of mortality and greater benefits on achieving negative blood cultures. However, the risk of AHF was not reduced. These findings extend our recent work using a propensity score matching approach to evaluate the treatment effects of warfarin on thromboembolic as well as hemorrhagic complications.^6^

The latest guidelines by the European Society of Cardiology for the management of endocarditis have recently been published in 2023.^5^ On the use of anticoagulation, the guidelines mentioned that patients with it does not appear to affect the “risk of stroke, cerebrovascular haemorrhage, or mortality at 10 weeks” in left‐sided IE, and thus it was reasonable to continue anticoagulation if there is a preexisting indication in the absence of other contraindications. A recent review article has appraised the evidence for and against initiation of anticoagulation in the context of IE, published before the European Society of Cardiology guidelines were available.^8^ The recommendation was that “interruption of anticoagulation without weighty reasons should not be considered.” Another recent systematic review of observational studies in 2024 further strengthened this recommendation by showing that anticoagulation therapy may reduce the risk of in‐hospital death after IE although it provided no benefits in stroke.^22^ The present study findings align with these previous data and provide additional evidence that anticoagulation using warfarin can be beneficial in terms of mortality by achieving negative blood cultures. Due to the lack of planned microbiological follow‐up in the current retrospective data, our analysis on achieving negative blood culture is exploratory in nature as such investigation depends on strain of pathogen, fever response, and local ward practices that varied in the real world over follow‐up period. Further prospective studies are needed to further investigate the mechanism behind warfarin and the achievement of negative culture results.

The role of warfarin in the management of IE remains controversial, because warfarin use was not associated with reducing risks of stroke compared with its counterparts.^22^, ^23^, ^24^ Currently, the compelling indication for warfarin in IE included patients with mechanical heart valves, ventricular assisted device, or atrial fibrillation with mitral stenosis.^25^, ^26^ In our previous study, in keeping with the current literatures, we found that the lowering of the mortality was not linked to lower risks of ischemic stroke events. Instead, the result hinted the possible role of vegetation size by anticoagulation.^27^ In this target trial emulation study, we confirmed the mortality benefits in IE after using warfarin.

In the pathophysiology of vegetation formation in patients with IE, the initial sterile platelet‐fibrin vegetation would be infected by microorganisms, which in turn, activate the coagulation pathway causing cumulation of the vegetation. Furthermore, the vegetation also provides an environment to protect the pathogen from the immune system clearance. Also, those particles may dislodge and load in the vascular beds causing thromboembolic complications including septic pulmonary embolism.^28^

The use of warfarin may have hastened the dissolution of vegetation, thus aiding the removal of the source of bacterial infection and thus infection resolution. As such, using the anticoagulants to reduce the vegetation may be explained through the achievement of culture negativity.^29^ This study introduces a novel approach of comparing the achievement of culture negative and AHF risks among patients with IE without warfarin. Although this study did not assess vegetation size, the shorter time to event and lower number of events for achieving a negative blood culture would support this hypothesis. Given the potential implications for infection control and hemodynamic stability, future studies should explore the role of anticoagulation in relation to septic shock and vegetation burden, ideally incorporating imaging and microbiological end points.

Partly owing to limited evidence, the role of anticoagulation in IE has been controversial. Previous observational studies on this topic investigated the role of anticoagulation in reducing possible neurological complications, with mixed results; In 1999, Tornos et al. first suggested that anticoagulant use contributed to higher mortality in prosthetic valve IE due to intracranial hemorrhage.^30^ Later in 2011, Syngg‐Martin et al. found that patients with ongoing warfarin treatment had lower risk of cerebrovascular complications such as ischemic infarction and cerebral infection.^31^ Garcia‐Cabrera et al. suggested an association between anticoagulant use and ischemic stroke or hemorrhage and recommended transitory discontinuation of anticoagulants in IE.^32^ In contrast, our previous study of 7054 patients with IE found no association between warfarin use and 90‐day risks of intracranial hemorrhage or ischemic stroke.^6^ However, certain IE conditions may be predisposed to elevated bleeding risk, such as presence of embolic infarctions, which may further undergo hemorrhagic transformation^8^; *S. Aureus* infections are also suggested to be associated with higher bleeding risk.^33^ Not all patients with IE are ideal candidates for warfarin and thus the decision to initiate anticoagulation remains individualized.

The use of antiplatelet agents in IE has similarly been controversial. It is theorized that inhibition of platelet activation prevents bacteria from attaching to damaged surfaces on the endocardium, thus reducing the risk of developing thromboembolism.^34^ In animal studies, aspirin was demonstrated to reduce bacterial titer and inhibit vegetation growth.^35^, ^36^ One observational study found antiplatelet use to be associated with lower 90‐day mortality but did not reduce cardioembolic events.^37^

Routinely collected, observational data have been used to estimate treatment effects in pharmacoepidemiological studies. However, even with propensity score matching or other matching methods, it is difficult to infer causality due to potential self‐inflicted bias from inappropriate study designs or analyses other than confounding control. The target trial emulation framework originally proposed by Hernán and Robins provides a way to reduce bias and emulating a clinical trial as closely as possible.^38^ This is particularly advantageous when a randomized controlled trial is not available or not feasible. In our case, randomization was emulated via cloning individuals and assigning each replicate to a treatment strategy, as the treatment is indistinctive at the baseline.^39^ This approach effectively minimized the time‐related bias without introducing selection bias. To deal with time‐varying confounders and enhance exchangeability over time, we calculated time‐varying inverse probability weight for each individual at each time.^39^ Using causal analytical approaches, we have been able to produce a comprehensive analysis with both absolute and relative risks given which may help better inform the clinical practice. Furthermore, with the trial emulation approach, a Consolidated Standards of Reporting Trials‐like flo wchart can be constructed, as is the case in randomized controlled trials.^40^ Overall, the current study has made substantial improvements in the design and analysis of observational data in answering this question as compared with existing research.^22^, ^27^

This study has several limitations. First, all diagnoses were ascertained from *ICD‐9* codes, and individual data adjudication was not possible. Nonetheless, the Clinical Data Analysis and Reporting System has been used extensively in research with demonstrable data completeness and coding accuracy.^41^, ^42^, ^43^ Specifically, there was incomplete identification of the causative pathogen. The causal agent was identified in only approximately one third of the IE episodes, which may introduce residual confounding due to the variation in prognosis and treatment response associated with different pathogens. Future studies with more comprehensive microbial data are warranted to better elucidate the heterogeneity of treatment effect of warfarin. Second, the dosage of warfarin, as well as whether therapeutic range was achieved, was not taken into consideration. Third, significant interethnic and interracial differences exist in the antithrombotic efficacy and bleeding risks of anticoagulants,^44^ meaning that our results, which are derived from mainly Han Chinese patients, may not be directly generalizable to patients of other ethnicities or races. Lastly, the causal inference from our study is based on the assumption of no unmeasured confounding, which is unverifiable in current setting. We observed that postbaseline cardiac surgeries and use of other cardiovascular medications such as antiplatelets and other anticoagulants are more common in the warfarin arm. Although we controlled for these factors using a time‐varying inverse probability weighting method, residual confounding may affect our analysis as these factors are likely to affect the prognosis of IE.

## CONCLUSIONS

This cohort study applying the target trial emulation framework found that warfarin use was associated with a significantly lower risk of mortality with benefits on achieving negative blood cultures but similar risk of AHF.

## Sources of Funding

This work was funded by the Tianjin Key Medical Discipline (Specialty) Construction Project (project number: TJYXZDXK‐029A) a Research Impact Fund from Hong Kong Metropolitan University (Project Reference No. RIF/2022/2.2). The funders played no role in any part of this study.

## Disclosures

None.

## Supporting information

Data S1–S2
Tables S1–S11
