# Supplementary material for: Effects of Warfarin on the Risks of Mortality, Acute Heart Failure, and Infection Resolution in Patients With Infective Endocarditis: A Target Trial Emulation
Source: J Am Heart Assoc. 2025 Jul 17;14(15):e041965. doi: 10.1161/JAHA.125.041965 (PMC12449976; doi:10.1161/JAHA.125.041965)
Supplement: Supplementary file 1 — Data S1–S2 Tables S1–S11 [file JAH3-14-e041965-s001.pdf]

SUPPLEMENTAL MATERIAL

Data S1. Directed acyclic graph of causal structure among study exposure, outcome, and confounders.

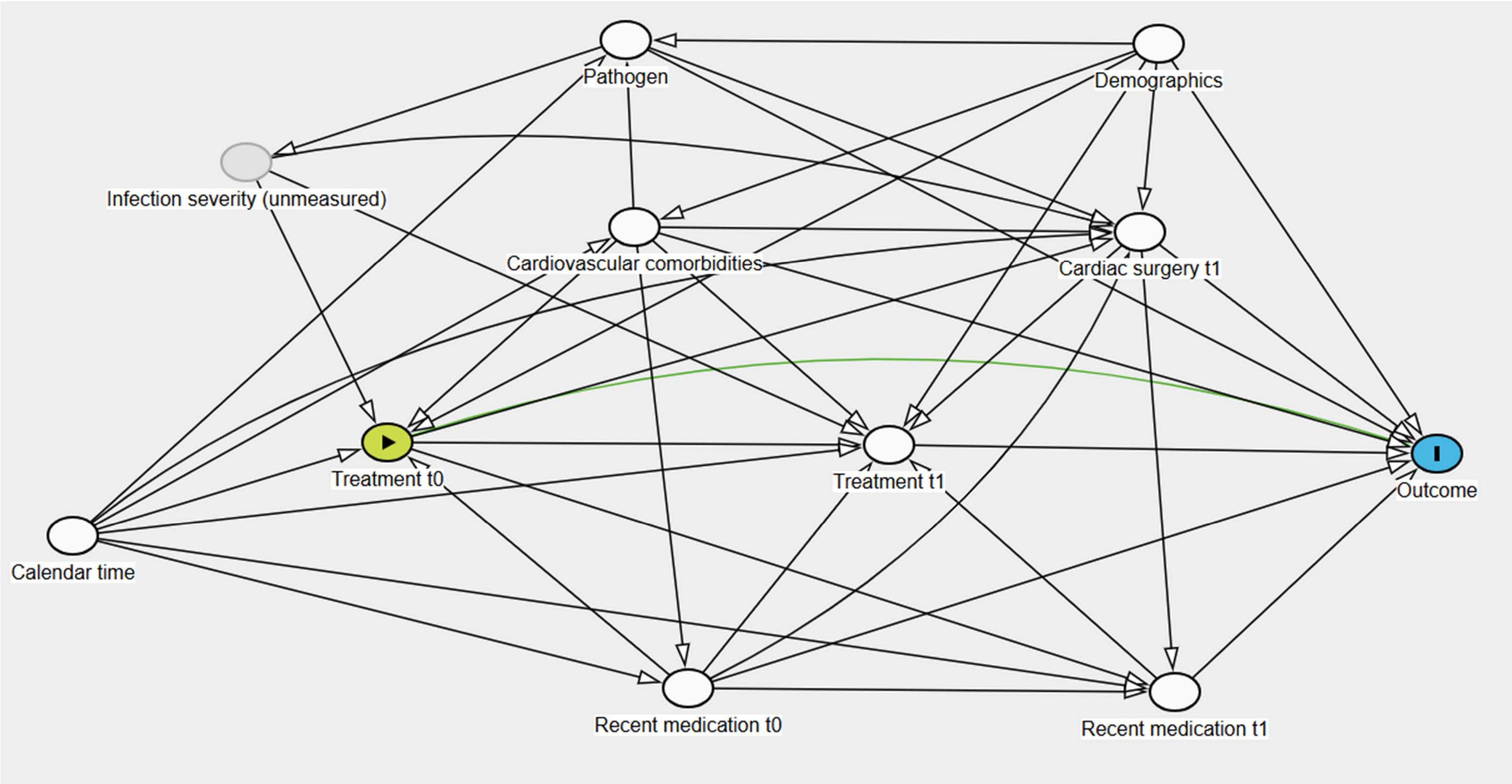

Directed acyclic graph (DAG) illustrating the assumed causal relationships between warfarin treatment at baseline (t0), subsequent warfarin treatment (t1), and the outcome (all-cause mortality, acute heart failure, and negative blood culture), accounting for measured and unmeasured covariates. For simplicity, only baseline variables and time-varying variables at t1 were illustrated. Calendar time, demographics, cardiovascular comorbidities, infection severity (unmeasured), pathogen, prior medication use, and cardiac surgery are considered potential confounders. The green arrow indicates the causal effect of interest from Treatment t0 to the Outcome.

Data S2. Details of the clone-censor-weight analysis.

The clone-censor-weight design is a three-step approach that is commonly used to emulate target pragmatic trials using observational data. As we explained in the main text and in Table S1, the trial emulation has two steps. First, we designed a pragmatic target trial that would answer the causal question of interest; then, we emulated this using data from CDARS. As there are intrinsic differences between observational data in real world and data from a randomised controlled trial settings (mainly due to the lack of randomised treatment assignment at the cohort entry), we would need certain study designs to allow a close emulation of the target trial, e.g., clone-censor-weight. The main advantage of this approach is in reduction of bias when the treatment status (i.e., the receipt of warfarin treatment) is indistinguishable at the baseline (i.e., the IE diagnosis).

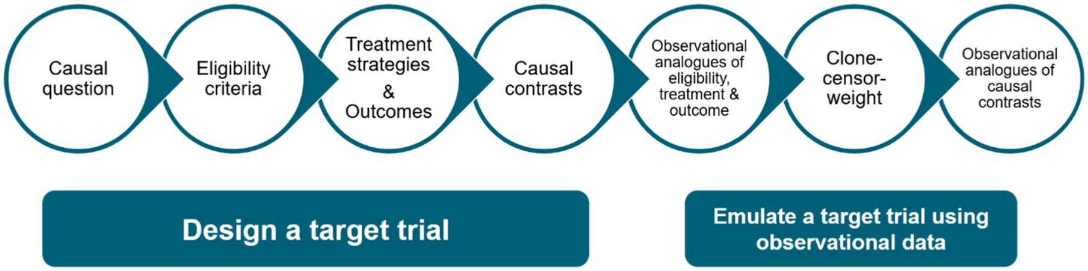

Schema of trial emulation with clone-censor-weight approach.

Step 1. Clone

From the dataset that contains all study variables of all eligible patients, we simply replicate this dataset to *N* copies (*N* equals to the number of treatment strategies one would like to compare in the target trial, in this case, *N*=2). One copy is assigned to the "initiating warfarin treatment 2 weeks within the infective endocarditis diagnosis" strategy and the other copy is assigned to the "no warfarin treatment" strategy.

Step 2. Censor

Because the treatment assignment is hypothetical, patient replicates are likely to deviate from the assigned treatment strategy in real world. Therefore, we censor the patient replicates upon treatment deviation. That is, if a patient replicate is assigned to initiating warfarin treatment strategy and has not done so within the 2-week grace period, they are censored after the grace period; if a patient replicate is assigned to no warfarin treatment strategy but initiate the treatment at any time during the follow-up, they are censored at the treatment initiation.

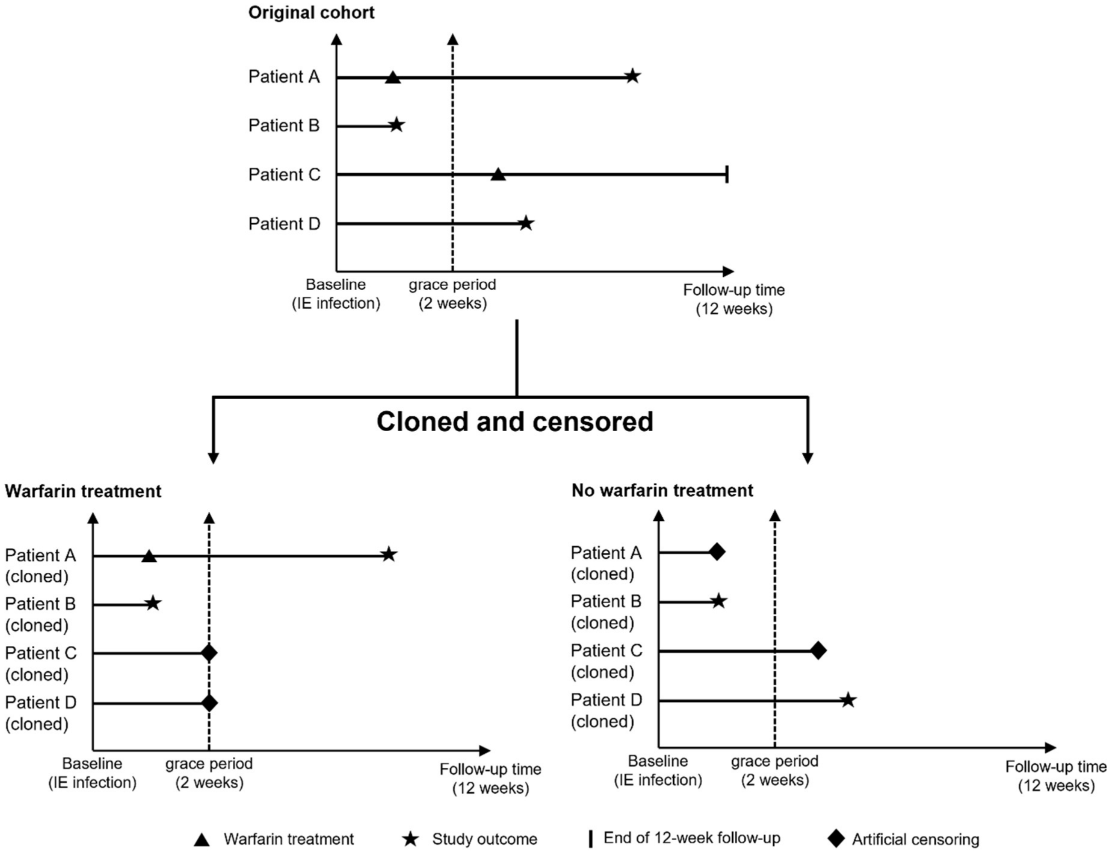

We further illustrate the cloning and censoring process using four hypothetical patients as the examples.

- Patient A received warfarin within the 2-week grace period and experienced a study outcome (e.g., death) during follow-up.

- Patient B had a study outcome within the grace period before receiving warfarin.
- Patient C received warfarin after the grace period and did not experience an outcome during the 12-week follow-up period therefore reached the end of follow-up.
- Patient D had a study outcome after the grace period and had not received any warfarin prior to this event.

All patients are cloned and assigned to both "warfarin treatment within 2-week" and "no warfarin treatment".

- Patient A assigned to warfarin arm is compatible with the assigned strategy and is therefore not artificially censored and followed until the study outcome; Patient A assigned to no warfarin arm was artificially censored at the warfarin treatment.
- Patient B is not censored in either treatment arm, and contributes the outcome to both treatment strategies.
- Patient C assigned to warfarin arm is artificially censored after the grace period; Patient C assigned to no warfarin arm is artificially censored at the warfarin treatment after the grace period.
- Patient D assigned to warfarin arm is artificially censored after the grace period; Patient D assigned to no warfarin arm is not artificially censored and followed until the study outcome.

Step 3. Weight

Because the artificial censoring in step 2 can induce attrition bias, we correct the bias by building an inverse probability of censoring weight, based on the probability of a patient (replicate) of being uncensored at each time. To do so, we first calculate the probability of a patient initiating warfarin treatment at each time interval based on their baseline characteristics.

This probability is calculated using logistic regression models. We fit two models, one for each treatment strategy arm, to allow treatment-covariate interactions. The first model, used for initiating warfarin treatment arm, was restricted to time interval (weekly) from t0 and t1; the second model, used for the no warfarin treatment arm used all observations from time interval t0 to t11. Then the inverse probability weight is built as

$$W_t^{A=1} = \frac{1}{1 - \prod_{k=0}^{t} \Pr(a_k = 0 | Y_{k-1} = 0, D_{k-1} = 0, \bar{a}_{k-1} = 0, V, \bar{L})}$$

$$W_t^{A=0} = \frac{1}{\prod_{k=0}^{t} \Pr(a_k = 0 | Y_{k-1} = 0, D_{k-1} = 0, \bar{a}_{k-1} = 0, V, \bar{L})}$$

where Y=outcome, D=death, A=treatment assignment, a=treatment initiation, V=baseline covariates, L=time-varying covariates, t=time interval since baseline

The $W_t^{A=1}$ is artificially kept as 1 for t0 and t1, and unchanged from t3 onwards. This is because during these time intervals, by definition, the artificial censoring cannot occur.

Step 4. Analysis

The replicated datasets with calculated weights are stacked and analysed with a weighted pooled logistic regression model. The model further included all baseline covariates, a treatment indicator, time (in its linear and quadratic terms), and the interaction terms between time (in its linear and quadratic terms) and treatment indicator. This model predicts the risk of study outcome for each participant under each treatment strategy at each time interval. We can use these predicted risks to compute the population-average cumulative risk (and risk difference) of the study outcome under treatment strategies. This can be interpreted as the causal estimand that we aim to answer under the assumption of exchangeability, positivity, consistency, and no model misspecification.

Table S1. Brief protocol of the pragmatic target trial and its emulation using data from CDARS.

Component	Target trial	Emulation in CDARS
Eligibility	Individuals (both sexes) aged 18 years or older diagnosed with infective endocarditis (IE) between 1st Jan 1997 and 31st Aug 2020. Patients will be excluded if they have previous heart failure, valvular replacement, or use of warfarin or any other uses of DOACs (rivaroxaban, dabigatran, apixaban, edoxaban, enoxaparin, fondaparinux, heparin) within 30 days prior to IE diagnosis.	Same as the target trial.
Treatment strategies	Warfarin treatment within 14 days of the IE diagnosis vs no warfarin treatment.	Same as the target trial.
Treatment assignment	Eligible individuals are randomly assigned to one of the two strategies.	Randomisation is emulated via cloning individuals and assigning each replicate to a treatment strategy.
Follow-up	For each individual, follow-up starts at the time of IE diagnosis and assignment to a strategy and ends at the occurrence of an outcome, death, 12 weeks after the index date, whichever comes first.	Same as the target trial.
Primary endpoint	All-cause mortality, acute heart failure, negative blood culture.	Same as the target trial.
Causal contrast	Intention-to-treat effect.<br>Per protocol effect.	Observational analogue of per protocol effect: effect of adhering to the strategies as specified under "Treatment strategies" during follow-up, i.e., patients clones assigned to the "warfarin treatment" arm are censored after Day 14 if they did not receive warfarin by Day 14 after the IE diagnosis; patient clones assigned to the "no warfarin treatment" arm are censored at any time during the follow-up if they receive warfarin treatment during the follow-up.
Statistical analysis	Intention-to-treat analysis.<br>Per protocol analysis: Individuals are artificially censored when they deviate from their assigned strategy	Same as per protocol analysis. We created an expanded dataset including 2 replicates for each included individual and assigned one replicate to each treatment strategy. We adjusted for the baseline covariates and assumed that adjustment for these variables was sufficient to adjust for informative censoring.

Table S2. Model coefficients for not receiving warfarin treatment by treatment arms for each study outcome.

Outcome	Death				AHF				Negative blood culture			
**Treatment arm**	**Warfarin arm**		**No warfarin arm**		**Warfarin arm**		**No warfarin arm**		**Warfarin arm**		**No warfarin arm**	
**Parameter**	**Estimate**	**Standard error**	**Estimate**	**Standard error**	**Estimate**	**Standard error**	**Estimate**	**Standard error**	**Estimate**	**Standard error**	**Estimate**	**Standard error**
Intercept	2.9532	0.9145	3.2160	0.6689	3.2247	0.9562	3.483	0.7657	0.8216	0.888	7.868	258.9
Week	0.0897	0.0986	0.7311	0.0413	0.0304	0.1037	0.7918	0.0466	-0.0853	0.1982	0.6631	0.1523
Week*week	0	.	-0.0521	0.00402	0	.	-0.0578	0.00454	0	.	-0.0362	0.0201
**Baseline**												
Age	0.0064	0.00374	0.0068	0.00315	0.00647	0.00396	0.00648	0.00346	0.00659	0.00702	0.00603	0.00722
Female sex	-0.1885	0.0462	-0.0738	0.0379	-0.1861	0.049	-0.0756	0.0423	-0.3071	0.086	-0.2775	0.0889
CCI	0.038	0.0396	0.1265	0.0371	0.0275	0.0422	0.1088	0.0405	0.0657	0.0789	0.0514	0.0809
Calendar 1	3.0047	0.4169	2.4623	0.2968	2.9003	0.4173	2.3053	0.2979	0.4374	0.6397	1.1155	0.8892
Calendar 2	-0.1689	0.1292	-0.1454	0.1046	-0.2556	0.1308	-0.214	0.1091	-0.3353	0.2316	-0.1672	0.259
Calendar 3	-0.4421	0.1272	-0.4458	0.1002	-0.5219	0.1284	-0.5547	0.1029	-0.3202	0.2006	-0.4141	0.2226
Calendar 4	-0.3745	0.1288	-0.2256	0.1039	-0.4312	0.1314	-0.2962	0.1088	0.00456	0.2229	-0.0918	0.2455
Calendar 5	-0.6403	0.1221	-0.4532	0.1006	-0.7398	0.1238	-0.5375	0.105	-0.2508	0.218	-0.3468	0.2398
Calendar 6	-0.2022	0.1335	-0.2111	0.1024	-0.2515	0.1383	-0.269	0.1097	0.024	0.2351	-0.06	0.2605
Calendar 7	-0.5854	0.1214	-0.4904	0.0935	-0.4165	0.1351	-0.2578	0.1146	0.5486	0.2698	0.2347	0.275
Hypertension	0.2266	0.0815	0.0159	0.0681	-0.182	0.0863	0.0585	0.0762	-0.2116	0.1651	-0.1848	0.167
Atrial fibrillation	-1.0821	0.0698	-0.8277	0.0656	1.063	0.0729	0.8317	0.0705	1.3064	0.1235	1.2788	0.1292
Diabetes mellitus	0.1817	0.0955	0.2004	0.0826	-0.1452	0.1005	-0.2282	0.0974	-0.0362	0.1833	-0.0294	0.1935
Intracranial haemorrhage	0.2125	0.1742	0.1021	0.1481	-0.1752	0.1778	-0.1509	0.1599	0.0427	0.2636	-0.0292	0.2939
Chronic kidney disease	0.4367	0.156	0.5583	0.1226	-0.4526	0.1707	-0.7895	0.1525	-0.2854	0.2721	-0.2237	0.2705
Vascular disease	0.0108	0.279	0.2262	0.3038	0.0938	0.2846	-0.2638	0.3684	0.1347	0.441	-0.0549	0.553
ACE inhibitors	-0.2067	0.0904	0.0772	0.0735	0.2789	0.0953	-0.029	0.0806	0.1654	0.1997	-0.079	0.2077
ARBs	0.1477	0.2601	-0.049	0.1599	-0.409	0.2848	-0.1603	0.201	-0.7666	0.5348	-0.7399	0.474
Beta-blockers	0.2209	0.1176	0.0443	0.08	-0.1001	0.1315	-0.0307	0.0935	-0.5473	0.2828	-0.3064	0.2898
Antiplatelets	-0.2899	0.1768	0.0483	0.1102	0.2765	0.1959	0.0957	0.1306	1.2262	0.595	0.1416	0.3934
NSAIDs	-0.0216	0.1199	-0.1104	0.0798	-0.0561	0.1334	-0.0072	0.0953	-0.3571	0.2449	-0.1645	0.2419
H2 receptor antagonists	0.1445	0.1084	0.1832	0.0692	-0.1713	0.1196	-0.1379	0.0821	-0.3783	0.2401	-0.4265	0.2391
PPIs	0.1625	0.1169	0.3239	0.0731	-0.1593	0.1435	-0.3775	0.0902	0.1707	0.3044	-0.3188	0.2544
SSRIs	-0.8525	0.6434	-0.3126	0.3064	0.1473	0.6408	-0.1144	0.4075	-	-		
Staphylococci	0.392	0.0667	0.3982	0.0553	-0.3253	0.0687	-0.3458	0.0611	-0.0458	0.0946	-0.1244	0.1014
Streptococci	0.3663	0.0786	0.2298	0.059	-0.3161	0.0817	-0.1913	0.0651	-0.2313	0.1288	-0.1439	0.1252
Enterococci	0.1346	0.1733	0.1538	0.138	-0.2976	0.2126	-0.3396	0.1808	0.1613	0.2336	0.1255	0.2427
HACEK	1.1865	0.7115	0.6858	0.2989	-1.0543	0.7124	-0.4739	0.302	-	-		
**Time-varying**												
ACE inhibitors	-0.0877	0.0978	-0.4095	0.0593	0.0728	0.1046	0.4147	0.067	0.1852	0.2143	0.3813	0.1951

ARBs	-0.5326	0.2308	-0.2916	0.1299	0.7686	0.251	0.392	0.1569	1.2213	0.4906	1.0472	0.4053
Beta-blockers	-0.311	0.1015	-0.0699	0.0621	0.2411	0.1163	0.0531	0.0751	0.4033	0.2547	0.1377	0.259
Antiplatelets	0.5708	0.1296	0.1552	0.0703	-0.5025	0.1425	-0.2113	0.0855	-1.4517	0.5455	-0.4463	0.3055
NSAIDs	0.1952	0.09	0.0921	0.0573	-0.1972	0.0976	-0.0785	0.0665	-0.1025	0.1667	-0.1048	0.1695
H2 receptor antagonists	-0.1752	0.0928	-0.2245	0.0502	0.1749	0.1021	0.186	0.0596	0.3678	0.2096	0.2597	0.197
PPIs	0.1303	0.0986	-0.29	0.0512	-0.211	0.1207	0.3464	0.0606	-0.2445	0.2779	0.1929	0.2149
SSRIs	0.4334	0.6118	0.1461	0.2477	0.1198	0.5883	0.0519	0.2877	-	-	-	-
Cardiac surgeries	-0.4147	0.0784	-0.8634	0.0443	0.2033	0.0953	0.8766	0.0511	0.2769	0.1669	0.6001	0.1433
DOACs	0.6082	0.2359	1.2219	0.3618	-0.6677	0.2624	-1.0276	0.3677	-0.1591	0.6151	-6.5652	258.9
Heparins	-1.4791	0.0592	-0.959	0.0458	1.5343	0.0628	1.0602	0.0516	1.5231	0.1171	1.2314	0.1274

AHF, acute heart failure; CCI, Charlson's Comorbidity Index; ACE, angiotensin-converting enzyme; ARB, angiotensin-II-receptor blocker; NSAID, non-steroidal anti-inflammatory drug; H2, histamine-2; PPI, proton pump inhibitor; SSRI, selective serotonin receptor inhibitor; DOAC, direct oral anticoagulant.

In the analysis of negative blood culture outcome, no patients had recent use of SSRI or HACEK group pathogen.

Table S3. Inverse probability weight distribution for different outcomes.

All-cause mortality				
	Before truncation		After truncation	
**Measurement**	**Warfarin**	**No Warfarin**	**Warfarin**	**No Warfarin**
50th percentile	1.00	1.08	1.00	1.08
75th percentile	1.46	1.15	1.46	1.15
95th percentile	19.54	1.56	19.54	1.56
99th percentile	43.84	3.27	22.30	3.27
Max	520.62	11892.59	22.30	22.30
Mean	4.61	1.61	3.37	1.20
SD	18.59	59.51	5.56	0.84
**AHF**				
	**Before truncation**		**After truncation**	
**Measurement**	**Warfarin**	**No Warfarin**	**Warfarin**	**No Warfarin**
50th percentile	1.06	1.20	1.06	1.20
75th percentile	1.23	1.31	1.23	1.31
95th percentile	23.01	1.87	23.01	1.87
99th percentile	48.93	3.79	25.74	3.79
Max	641.42	21670.77	25.74	25.74
Mean	5.03	2.09	3.51	1.34
SD	22.98	110.91	6.33	0.83
**Negative blood culture**				
	**Before truncation**		**After truncation**	
**Measurement**	**Warfarin**	**No Warfarin**	**Warfarin**	**No Warfarin**
50th percentile	1.16	1.25	1.16	1.25
75th percentile	1.23	1.41	1.23	1.41
95th percentile	2.98	3.26	2.98	3.26
99th percentile	29.62	26.47	26.80	26.47
Max	1581.82	31.99	26.80	26.47
Mean	4.49	2.18	1.99	2.17
SD	58.78	4.27	4.06	4.19

SD, standard deviation.

Table S4. Baseline characteristics of the study cohorts after the grace period, before and after weighting.

	Before weighting			After weighting		
	**Warfarin – after the grace period (n=429)**	**No Warfarin – after the grace period (n=4,299)**	**SMD**	**Warfarin – after the grace period (n=5,705)**	**No Warfarin – after the grace period (n=5,031)**	**SMD**
**Age in years (SD)**	56.7 (16.5)	55.5 (19.2)	0.07	55.4 (49.5)	55.1 (20.4)	0.01
**Male sex (%)**	252 (58.7)	2,881 (67.0)	-0.62	3821 (67)	3279 (65.2)	-0.04
**CCI (SD)**	1.7 (1.9)	1.6 (2.1)	0.03	1.6 (6.3)	1.6 (2.2)	0.05
**Calendar year (%)**			0.66			0.11
1997-1999	2 (0.3)	751 (17.5)		710 (12.4)	757 (15)	
2000-2002	47 (7.6)	571 (13.3)		762 (13.4)	655 (13)	
2003-2005	54 (9.7)	505 (11.8)		813 (14.3)	605 (12)	
2006-2008	55 (9.8)	505 (11.8)		720 (12.6)	591 (11.7)	
2009-2011	64 (12.0)	470 (10.9)		626 (11)	614 (12.2)	
2012-2014	50 (9.6)	471 (11.0)		553 (9.7)	563 (11.2)	
2015-2017	79 (12.9)	532 (12.4)		837 (14.7)	641 (12.7)	
2018-2020	78 (13.6)	494 (11.5)		684 (12)	606 (12.1)	
**Comorbidities (%)**						
Hypertension	61 (14.2)	591 (13.8)	0.01	974 (17.1)	669 (13.3)	0.11
Atrial fibrillation	90 (20.1)	169 (3.9)	0.53	238 (4.2)	336 (6.7)	-0.11
Diabetes mellitus	33 (7.7)	395 (9.2)	-0.05	486 (8.5)	436 (8.7)	-0.01
Intracranial haemorrhage	9 (2.1)	89 (2.1)	0.00	160 (2.8)	99 (2)	0.06
Chronic kidney disease	11 (2.6)	189 (4.4)	-0.10	437 (7.7)	204 (4.1)	0.15
Vascular disease	2 (0.5)	25 (0.6)	-0.02	16 (0.3)	27 (0.5)	-0.04
**Recent medications (%)**						
ACE inhibitors	55 (12.8)	310 (7.2)	0.19	565 (9.9)	389 (7.7)	0.08
ARBs	17 (4.0)	89 (2.1)	0.11	76 (1.3)	110 (2.2)	-0.06
Beta-blockers	73 (17.0)	377 (8.8)	0.25	656 (11.5)	458 (9.3)	0.07
Antiplatelets	73 (17.0)	422 (9.8)	0.21	935 (16.4)	528 (10.5)	0.17
NSAIDs	94 (21.9)	642 (14.9)	0.18	1,227 (21.5)	795 (15.8)	0.15
H2 receptor antagonists	83 (13.6)	527 (12.3)	0.20	800 (14)	630 (12.5)	0.04
PPIs	55 (12.8)	448 (10.4)	0.07	782 (13.7)	537 (10.7)	0.09
SSRIs	4 (0.9)	38 (0.9)	0.01	21 (0.4)	45 (0.9)	-0.07
**Pathogen from blood culture (%)**						
Staphylococci	42 (9.8)	824 (19.2)	-0.27	1067 (18.7)	879 (17.5)	0.03
Streptococci	30 (7.0)	628 (14.6)	-0.24	567 (9.9)	679 (13.5)	-0.11
Enterococci	7 (1.6)	87 (2.0)	-0.03	121 (2.1)	102 (2)	0.01
HACEK	0 (0)	35 (0.8)	-0.13	0 (0)	36 (0.7)	-0.12

SD, standard deviation; CCI, Charlson's Comorbidity Index; ACE, angiotensin-converting enzyme; ARB, angiotensin-II-receptor blocker; NSAID, non-steroidal anti-inflammatory drug; H2, histamine-2; PPI, proton pump inhibitor; SSRI, selective serotonin receptor inhibitor.

Table S5. Proportion of patients with time-varying covariates at each week of follow-up.*

	**Warfarin**											**No warfarin**										
**Time-varying covariates**	Week 1	Week 2	Week 3	Week 4	Week 5	Week 6	Week 7	Week 8	Week 9	Week 10	Week 11	Week 1	Week 2	Week 3	Week 4	Week 5	Week 6	Week 7	Week 8	Week 9	Week 10	Week 11
ACE inhibitors	6.8%	15.9%	17.0%	17.1%	15.5%	14.4%	12.3%	11.4%	10.7%	11.6%	11.1%	6.4%	7.4%	8.3%	8.2%	8.1%	8.2%	8.2%	7.9%	7.5%	7.0%	6.4%
ARBs	2.9%	6.1%	6.4%	6.7%	5.8%	5.6%	5.4%	5.7%	6.0%	6.5%	6.0%	2.7%	2.7%	2.9%	3.0%	2.9%	2.9%	2.6%	2.5%	2.4%	2.3%	2.1%
Beta-blockers	14.4%	33.1%	35.1%	36.0%	34.1%	30.4%	29.7%	29.6%	25.7%	24.9%	25.6%	13.2%	14.1%	14.1%	14.6%	13.3%	12.8%	12.5%	11.7%	11.0%	10.7%	9.9%
Antiplatelets	16.0%	24.7%	25.0%	25.5%	18.4%	14.1%	12.5%	12.7%	12.0%	12.6%	11.8%	15.5%	15.5%	15.2%	13.4%	13.4%	12.7%	12.%	11.6%	10.8%	10.3%	9.6%
NSAIDs	13.9%	13.8%	16.3%	17.4%	14.3%	12.2%	11.5%	9.7%	8.2%	7.5%	6.5%	14.2%	15.7%	16.9%	16.9%	14.3%	12.8%	11.7%	10.8%	9.4%	8.4%	7.6%
H2 receptor antagonists	21.9%	35.2%	36.8%	37.4%	32.1%	26.8%	23.3%	23.4%	21.5%	21.4%	19.9%	21.4%	22.8%	23.4%	23.4%	21.2%	19.3%	18.5%	18.1%	16.7%	15.4%	13.4%
PPIs	19.9%	32.4%	34.4%	35.2%	35.3%	34.1%	29.7%	27.6%	26.2%	25.9%	25.1%	19.7%	22.0%	22.7%	23.1%	21.7%	20.1%	18.8%	17.4%	16.1%	15.1%	13.5%
SSRIs	1.1%	1.2%	1.2%	1.2%	1.0%	1.2%	1.2%	0.8%	0.8%	0.8%	0.8%	1.1%	1.2%	1.3%	1.3%	1.4%	1.5%	1.4%	1.4%	1.3%	1.4%	1.3%
Cardiac surgeries	8.0%	26.1%	27.4%	28.1%	22.5%	14.8%	11.8%	11.0%	9.0%	8.0%	0.7%	7.6%	11.6%	14.7%	15.6%	14.6%	12.3%	9.4%	7.5%	6.2%	6.0%	5.2%
DOACs	0.7%	2.8%	3.1%	3.3%	2.9%	2.2%	1.7%	1.2%	1.3%	1.5%	1.3%	0.6%	0.7%	0.8%	0.9%	0.8%	0.6%	0.7%	0.7%	0.6%	0.7%	0.7%
Heparins	10.0%	66.9%	70.1%	72.1%	62.1%	37.7%	25.3%	22.9%	20.0%	16.1%	14.1%	6.5%	7.0%	7.0%	7.5%	5.5%	4.2%	3.6%	3.2%	2.6%	2.1%	1.6%

ACE, angiotensin-converting enzyme; ARB, angiotensin-II-receptor blocker; NSAID, non-steroidal anti-inflammatory drug; H2, histamine-2; PPI, proton pump inhibitor; SSRI, selective serotonin receptor inhibitor.

*All covariates were measured at the start of the weekly intervals with a look-back window of 30 days.

Table S6. Sensitivity analysis: point estimates of 12-week absolute risks, risk differences, risk ratios, and hazard ratios for all-cause mortality, AHF, and negative blood culture under initiating warfarin and versus no warfarin, weights truncation at 99.5th percentile.

Treatment	No. of patients	No. of patient-weeks	No. of outcomes	12-week absolute risk (%)	12-week risk difference (%)	12-week risk ratio	Hazard ratio
**All-cause mortality**							
Warfarin	5,121	14,140	424	17.3	-5.6	0.76	0.75
No Warfarin	5,121	46,105	1,005	22.9	Reference	Reference	Reference
**AHF**							
Warfarin	5,121	12,837	592	22.5	1.8	1.09	1.09
No Warfarin	5,121	42,661	889	20.7	Reference	Reference	Reference
**Negative blood culture**							
Warfarin	3,662	5,155	2,829	96.4	11.5	1.14	1.17
No Warfarin	3,662	8,275	3,201	84.9	Reference	Reference	Reference

CI, confidence interval; AHF, acute heart failure.

Table S7. Sensitivity analysis: point estimates of 12-week absolute risks, risk differences, risk ratios, and hazard ratios for all-cause mortality, AHF, and negative blood culture under initiating warfarin and versus no warfarin, varied grace period.

Treatment	No. of patients	No. of patient-weeks	No. of outcomes	12-week absolute risk (%)	12-week risk difference (%)	12-week risk ratio	Hazard ratio
1-week grace period							
**All-cause mortality**							
Warfarin	5,121	19,279	557	17.2	-7.0	0.71	0.80
No Warfarin	5,121	46,105	1,005	24.2	Reference	Reference	Reference
**AHF**							
Warfarin	5,121	17,350	677	21.6	1.0	1.05	1.05
No Warfarin	5,121	42,661	889	20.6	Reference	Reference	Reference
**Negative blood culture**							
Warfarin	3,662	5,206	2,838	96.2	11.1	1.13	1.16
No Warfarin	3,662	8,275	3,201	85.0	Reference	Reference	Reference
3-week grace period							
**All-cause mortality**							
Warfarin	5,121	8,351	233	13.1	-8.7	0.61	0.79
No Warfarin	5,121	46,105	1,005	21.8	Reference	Reference	Reference
**AHF**							
Warfarin	5,121	7,789	432	18.9	-1.4	0.93	1.05
No Warfarin	5,121	42,661	889	20.3	Reference	Reference	Reference
**Negative blood culture**							
Warfarin	3,662	3,828	2,295	97.3	12.3	1.14	1.11
No Warfarin	3,662	8,275	3,201	85.1	Reference	Reference	Reference

CI, confidence interval; AHF, acute heart failure.

Table S8. Sensitivity analysis: point estimates of 12-week absolute risks, risk differences, risk ratios, and hazard ratios for all-cause mortality, AHF, and negative blood culture under initiating warfarin and versus no warfarin, doubly robust estimation.

Treatment	No. of patients	No. of patient-weeks	No. of outcomes	12-week absolute risk (%)	12-week risk difference (%)	12-week risk ratio	Hazard ratio
**All-cause mortality**							
Warfarin	5,121	14,140	424	17.2	-5.3	0.76	0.76
No Warfarin	5,121	46,105	1,005	22.6	Reference	Reference	Reference
**AHF**							
Warfarin	5,121	12,837	592	22.8	2.7	1.13	1.13
No Warfarin	5,121	42,661	889	20.1	Reference	Reference	Reference
**Negative blood culture**							
Warfarin	3,662	5,155	2,829	96.7	4.1	1.05	1.08
No Warfarin	3,662	8,275	3,201	92.7	Reference	Reference	Reference

CI, confidence interval; AHF, acute heart failure.

Table S9. Sensitivity analysis: point estimates of 12-week absolute risks, risk differences, risk ratios, and hazard ratios for AHF (post-operative use of inotropes and vasopressors excluded) under initiating warfarin and versus no warfarin.

Treatment	No. of patients	No. of patient-weeks	No. of outcomes	12-week absolute risk (%)	12-week risk difference (%)	12-week risk ratio	Hazard ratio
**AHF**							
Warfarin	5,121	12,964	559	18.4	0.3	1.02	1.02
No Warfarin	5,121	42,830	812	18.1	Reference	Reference	Reference

CI, confidence interval; AHF, acute heart failure.

Table S10. Sensitivity analysis: point estimates of 12-week absolute risks, risk differences, risk ratios, and hazard ratios for AHF (any use of inotropes and vasopressors excluded) under initiating warfarin and versus no warfarin.

Treatment	No. of patients	No. of patient-weeks	No. of outcomes	12-week absolute risk (%)	12-week risk difference (%)	12-week risk ratio	Hazard ratio
**AHF**							
Warfarin	5,121	12,837	366	10.3	-1.7	0.87	0.87 (0.71 to 1.06)
No Warfarin	5,121	42,661	527	12.0	Reference	Reference	Reference

CI, confidence interval; AHF, acute heart failure.

Table S11. Sensitivity analysis: point estimates of 12-week absolute risks, risk differences, risk ratios, and hazard ratios for all-cause mortality, AHF, and negative blood culture under initiating warfarin and versus no warfarin, stratified by calendar time.

Treatment	No. of patients	No. of patient-weeks	No. of outcomes	12-week absolute risk (%)	12-week risk difference (%)	12-week risk ratio	Hazard ratio (95% CI)
**All-cause mortality**							
Year 1997 to 2008							
Warfarin	2,688	6,751	212	17.6	-2.1	0.89	0.90 (0.65 to 1.24)
No Warfarin	2,688	25,586	478	19.8	Reference	Reference	Reference
Year 2009 to 2020							
Warfarin	2,433	7,389	212	14.5	-11.0	0.57	0.55 (0.40 to 0.74)
No Warfarin	2,433	20,519	527	25.4	Reference	Reference	Reference
**AHF**							
Year 1997 to 2008							
Warfarin	2,688	6,624	103	8.5	4.6	1.54	1.54 (0.92 to 2.53)
No Warfarin	2,688	25,183	159	13.1	Reference	Reference	Reference
Year 2009 to 2020							
Warfarin	2,433	6,213	489	30.9	-3.2	0.91	0.89 (0.73 to 1.09)
No Warfarin	2,433	17,478	730	34.1	Reference	Reference	Reference
**Negative blood culture**							
Year 1997 to 2008							
Warfarin	1,460	2,242	975	92.5	18.3	1.25	1.25 (1.06 to 1.47)
No Warfarin	1,460	4,349	1,181	74.2	Reference	Reference	Reference
Year 2009 to 2020							
Warfarin	2,202	2,913	1,854	99.4	3.0	1.03	1.05 (0.97 to 1.14)
No Warfarin	2,202	3,926	2,020	96.5	Reference	Reference	Reference

CI, confidence interval; AHF, acute heart failure.
